# Early outcomes after small incision lenticule extraction and photorefractive keratectomy for correction of high myopia

**DOI:** 10.1038/srep32820

**Published:** 2016-09-07

**Authors:** Tommy C. Y. Chan, Marco C. Y. Yu, Alex Ng, Zheng Wang, George P. M. Cheng, Vishal Jhanji

**Affiliations:** 1 Department of Ophthalmology and Visual Sciences, The Chinese University of Hong Kong; 2 Hong Kong Eye Hospital, Kowloon, Hong Kong; 3 Department of Mathematics and Statistics, Hang Seng Management College, Hong Kong; 4 Department of Ophthalmology, The University of Hong Kong, Hong Kong; 5 Zhongshan Ophthalmic Center, Sun Yat-sen University, Guangzhou, China Hong; 6 Kong Laser Eye Center, Hong Kong.

## Abstract

We prospectively compared visual and refractive outcomes in patients with high myopia and myopic astigmatism after small-incision lenticule extraction (SMILE) and photorefractive keratetctomy (PRK) with mitomycin C. Sixty-six eyes of 33 patients (mean age, 29.7 ± 5.6 years) were included (SMILE: 34 eyes, PRK 32 eyes). Preoperatively, no significant difference was noted in manifest spherical equivalent (p = 0.326), manifest sphere (p = 0.277), and manifest cylinder (p = 0.625) between both groups. At 1 month, there were significant differences in logMAR uncorrected distance visual acuity, efficacy index and manifest refraction spherical equivalent between SMILE and PRK (p ≤ 0.029). At 6 months, the logMAR corrected distance visual acuity (p = 0.594), logMAR uncorrected visual acuity (p = 0.452), efficacy index (p = 0.215) and safety index was (p = 0.537) was comparable between SMILE and PRK. Significant differences were observed in postoperative manifest spherical equivalent (p = 0.044) and manifest cylinder (p = 0.014) between both groups. At the end of 6 months, 100% of the eyes in SMILE group and 69% of the eyes in PRK group were within ±0.50 D of the attempted cylindrical correction. The postoperative difference vector, magnitude of error and absolute angle of error were significantly smaller after SMILE compared to PRK (p ≤ 0.040) implying a trend towards overcorrection of cylindrical correction following PRK.

The traditional management options for correction of high myopia include laser *in situ* keratomileusis (LASIK) and photorefractive keratetctomy (PRK). The correction of high myopia is challenging owing to the associated risk of treatment regression and corneal ectasia. Although LASIK provides quick visual rehabilitation, PRK has been shown to offer early stabilization of cornea while allowing for correction of higher myopic correction compared to LASIK^1^. Moreover, corneal biomechanics are affected less by flapless techniques than it is with LASIK^2^. Small-incision lenticule extraction (SMILE) is a flapless, all femtosecond refractive surgery technique in which a corneal stromal lenticule is fashioned using femtosecond laser and removed through a small peripheral corneal incision^3^,^4^. Previous studies have demonstrated that SMILE is safe and effective for management of myopia and myopic astigmatism^5^,^6^,^7^,^8^. A retrospective study comparing SMILE and laser-assisted subepithelial keratectomy showed that changes in corneal hysteresis and corneal resistance factor induced by SMILE are more predictable than those induced by laser-assisted subepithelial keratectomy^9^. However, there is a lack of comparative data on visual and refractive outcomes between SMILE and PRK. The current aims to compare the visual and refractive outcomes between SMILE and PRK with mitomycin C for high myopic eyes with low to moderate (<3 D) astigmatism.

## Results

Sixty-six eyes of 33 patients with a mean age of 29.7 ± 5.6 years were included (SMILE: 34 eyes, PRK 32 eyes). Preoperatively, there was no significant difference in manifest spherical equivalent (p = 0.326), manifest sphere (p = 0.277), and manifest cylinder (p = 0.625) between both groups (Table 1). Corneas were thinner in eyes receiving PRK (525.1 ± 22.7 μm) compared to SMILE (545.1 ± 20.6 μm) (p = 0.012). All surgeries were uneventful without any intraoperative complications. No postoperative complications, such as wound dehiscence, inflammation and infection were observed in any patient. Mild subepithelial corneal haze (grade 0.5) was seen in all eyes after PRK, but resolved by three months postoperatively.

At 1 month, there were significant differences in UDVA, efficacy index and manifest refraction spherical equivalent between SMILE and PRK (p ≤ 0.029) (Table 1). The logMAR CDVA and safety index remained comparable between groups (p ≥ 0.188).

**Figure 1:**
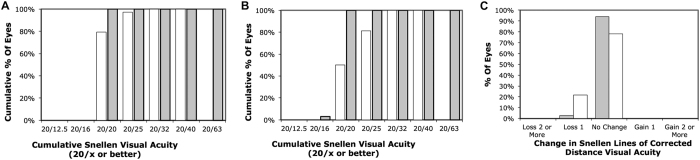
Visual outcomes by 6 months: Cumulative percentage of eyes attaining specified cumulative levels of postoperative uncorrected distance visual acuity (UDVA) for small-incision lenticule extraction (**A**) and photorefractive keratectomy (**B**) (grey = preoperative, white = postoperative), and change in preoperative and postoperative CDVA (grey = SMILE, white = PRK).

**Figure 2:**
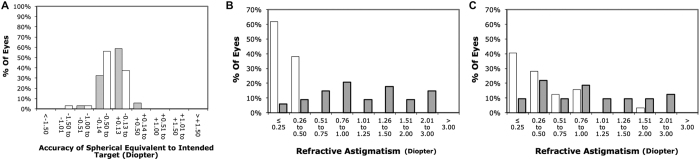
Refractive outcomes by 6 months: Percentages of eyes within different diopter ranges of the intended correction in spherical equivalent (**A**) (grey = SMILE, white = PRK), and percentage of eyes attaining specified levels of astigmatism before and after small-incision lenticule extraction (**B**) and photorefractive keratectomy (**C**) (grey = preoperative, white = postoperative).

**Figure 3:**
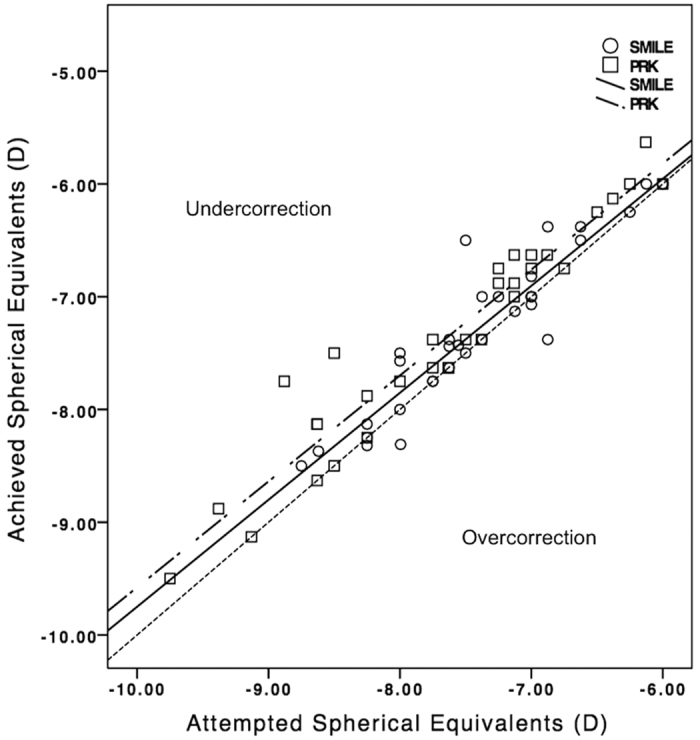
Attempted versus achieved manifest spherical equivalent correction for small-incision lenticule extraction and photorefractive keratectomy by 6 months.

At 6 months, the logMAR CDVA was 0.021 ± 0.055 and 0.013 ± 0.049 following SMILE and PRK, respectively (p = 0.594). The corresponding logMAR UDVA was 0.044 ± 0.058 and 0.060 ± 0.093 (p = 0.452). The efficacy index was 0.92 ± 0.12 after SMILE and 0.86 ± 0.19 after PRK (p = 0.215). The corresponding safety index was 0.96 ± 0.10 and 0.94 ± 0.13 (p = 0.537) (Table 1). The distribution of UDVA and CDVA following SMILE and PRK was presented in Fig. 1. Significant differences were observed in postoperative manifest spherical equivalent (p = 0.044) and manifest cylinder (p = 0.014) between SMILE and PRK (Table 1). At the end of 6 months, 97% (33 eyes) and 94% were within ±0.50 D of the attempted spherical equivalent correction after SMILE and PRK respectively (Fig. 2). As for manifest cylinder, 100% (34 eyes) and 69% (22 eyes) were within ±0.50 D of the attempted cylindrical correction after SMILE and PRK, respectively (Fig. 2). Scatterplots of achieved versus attempted spherical equivalent are shown in Fig. 3.

### Vector analysis

**Figure 4:**
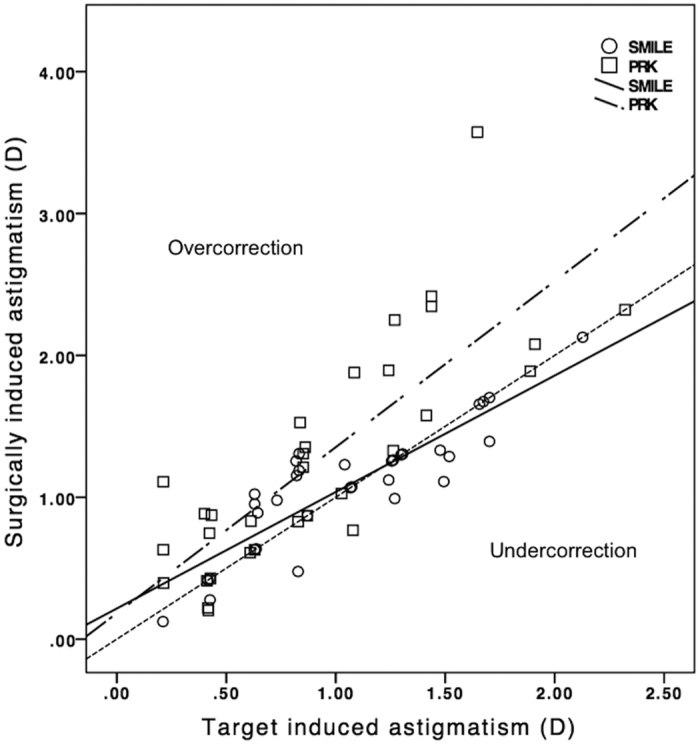
Target induced astigmatism vector versus surgically induced astigmatism vector for small-incision lenticule extraction and photorefractive keratectomy by 6 months.

**Figure 5:**
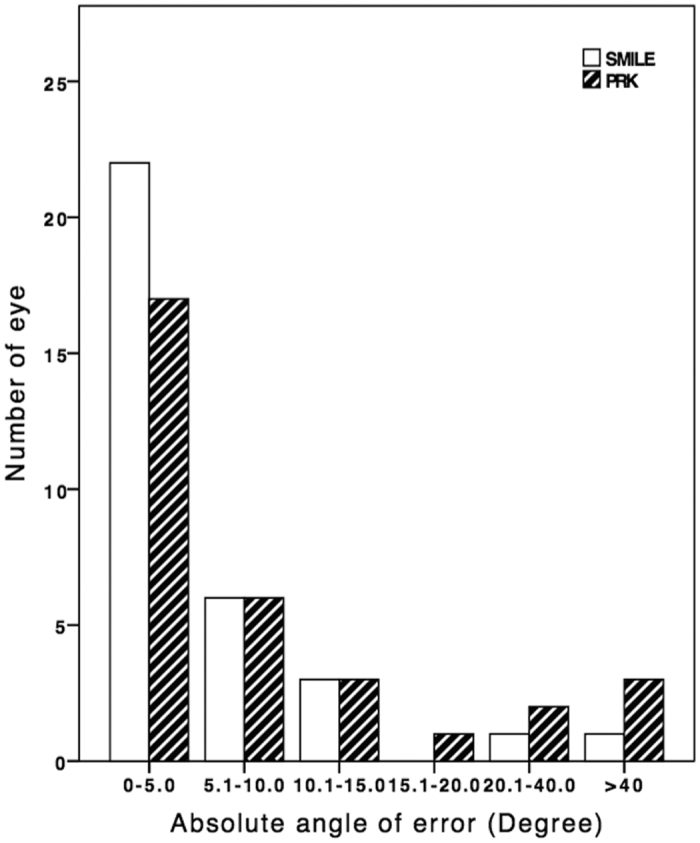
Distribution of absolute angle of error for small-incision lenticule extraction and photorefractive keratectomy by 6 months.

**Figure 6:**
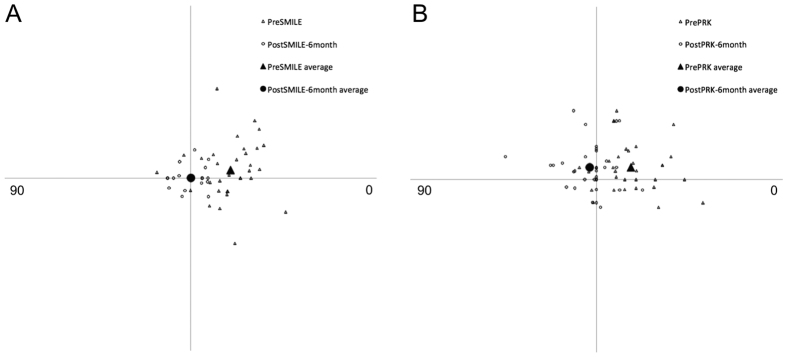
Double-angle plots for preoperative target induced astigmatism (open triangle) and postoperative difference vector at postoperative 6 months (open circle) after small-incision lenticule extraction (**A**) and photorefractive keratectomy (**B**).

The vector analysis results using the 6-month refractive data are shown in Table 2. There was no significant difference in the arithmetic mean of TIA and SIA between SMILE and PRK (p > 0.235). Scatterplots of SIA versus TIA for both groups are shown in Fig. 4. The postoperative DV, ME and absolute AE were significantly smaller after SMILE compared to PRK (p ≤ 0.040). The spread of absolute AE was higher for increasing TIA in both groups (Fig. 4). The distributions of absolute AE are presented in Fig. 5. Double-angle plots for both procedures are shown in Fig. 6.

**Figure 7:**
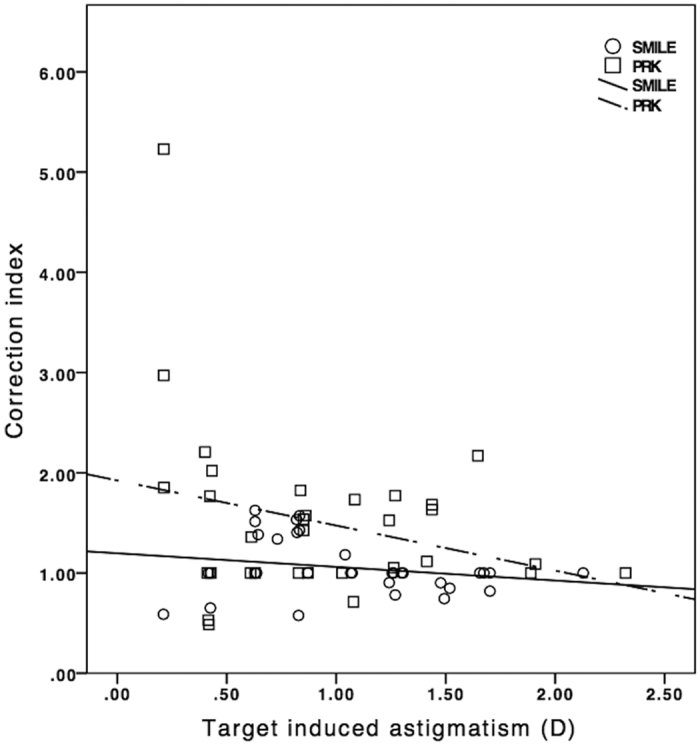
Correction index versus surgically induced astigmatism vector for small-incision lenticule extraction and photorefractive keratectomy by 6 months.

**Figure 8:**
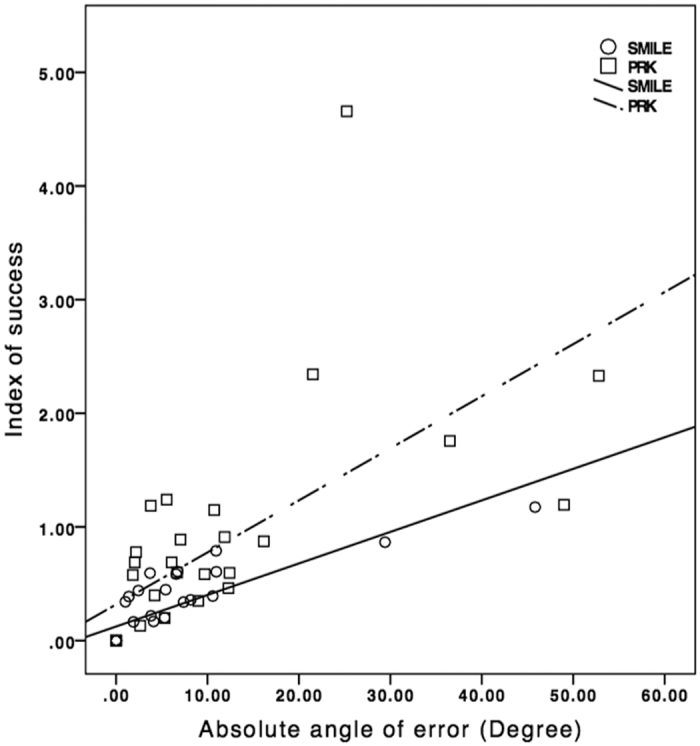
Index of success versus absolute angle of error for small-incision lenticule extraction and photorefractive keratectomy by 6 months.

At 6 months, the correction index (CI), defined as the ratio of SIA to TIA, was 1.02 (95% CI: 1.00 to 1.04) in SMILE and 1.34 (95% CI: 1.29 to 1.40) in PRK. Figure 7 shows the scatterplots of CI plotted against TIA in both groups. Index of success (IS), defined as DV divided by TIA, was 0.13 (95% CI: 0.12 to 0.15) and 0.48 (95% CI: 0.42 to 0.53) in SMILE and PRK, respectively. Significant correlation was found between absolute AE and IS for both procedures (r = 0.906 for SMILE, r = 0.827 for PRK; p < 0.001) (Fig. 8). Flattening index (FI), defined as ratio of the amount of astigmatism reduction achieved by the effective proportion of the SIA at the intended meridian to TIA, was 0.78 (95% CI: 0.72 to 0.83) in SMILE and 0.90 (95% CI: 0.83 to 0.97) in PRK (Table 2).

## Discussion

The current study showed that SMILE was more effective in low to moderate astigmatic correction compared to PRK at 6 months. Although both procedures were found to be similar in spherical correction and safety, visual rehabilitation was quicker after SMILE compared to PRK. This could represent a differential healing response between both procedures^10^. Previous studies investigating astigmatic correction following PRK reported that refractive predictability was worse in PRK for astigmatic treatment compared to spherical correction alone^11^,^12^. PRK induced unintentional astigmatism, which increased with the amount of myopic treatment^11^. The effectiveness was reduced for correction of low astigmatism^13^,^14^. In our study, we observed a trend towards overcorrection of cylindrical value despite a slight undercorrection of spherical equivalent following PRK. According to vector analysis, PRK had a CI of 1.34 signifying overcorrection, which appeared to increase with lower TIA. Likewise, Katz *et al*. reported a CI of 1.25 for PRK in eyes with myopic astigmatism^15^.

We found that SMILE was more effective than PRK for correction of low (1.0 to 1.5 D) myopic astigmatism. Slight overcorrection was observed in eyes with low astigmatism, while slight undercorrection was observed in eyes with high astigmatism. Ivarsen and Hjortdal reported that undercorrection of astigmatism increased with attempted treatment following SMILE^16^. Undercorrection of astigmatic error has been reported in previous studies comparing SMILE to other corneal refractive surgeries^8^,^17^,^18^,^19^. The CI was very close to 1.0 for SMILE in our cohort, representing accurate astigmatic correction. It appeared to remain stable with increasing TIA for low to moderate astigmatic correction. Similar to PRK, we observed a slight undercorrection of spherical equivalent after SMILE. Our results match the findings of a recent large study demonstrating predictable treatment outcomes of up to 3.0 D of astigmatism with SMILE^20^. In another retrospective study comparing SMILE and laser assisted subepithelial keratectomy (LASEK), overcorrection and undercorrection of astigmatism was noted following SMILE and LASEK, respectively^17^. At 6 months postoperatively, the reported CI (1.05) for SMILE was similar to our results.

Overall, SMILE demonstrated better astigmatic correction compared to PRK. The residual spherical equivalent and astigmatism postoperatively were lower after SMILE. The observed difference in astigmatic correction between both procedures was likely influenced by the difference in absolute AE, which indicated alignment of treatment. High correlation between absolute AE and IS has been reported as well as demonstrated in the current study^21^. The absolute AE was significantly lower for SMILE compared to PRK in our cohort. Vector analysis demonstrates that the proportion of flattening effect is halved when treatment is misaligned by 30° 22. This emphasizes the importance of treatment alignment for successful correction of astigmatism, in addition to the treatment magnitude. Correction of low astigmatism is particularly a concern because of a higher tendency towards larger axis deviations upon vector analysis^8^,^19^,^23^. The spread of absolute AE was much wider for correction of low astigmatism for both procedures. Another possible reason for the difference between SMILE and PRK is better centration in SMILE that is maintained via continuous suction during the whole procedure. Comparative analysis between SMILE and LASIK reported less decentration in SMILE^24^. This may affect the overall alignment of astigmatic treatment.

It is possible that differential healing response between SMILE and PRK could be potential reasons for treatment misalignment. PRK utilizes ultraviolet light from excimer laser to reshape the corneal curvature through direct photoablation. SMILE uses femtosecond laser in near infrared spectrum to achieve the cutting effect within the cornea through photodisruption. The amount of energy delivered to the cornea was much lower with femtosecond laser compared to excimer laser^25^. The main advantage of femtosecond laser compared to excimer laser is its high cutting precision with minimal collateral damage and associated cellular and structural changes^10^. The response of corneal epithelium may also be different between SMILE and PRK, which required epithelial removal of the treatment zone. This is important because epithelial hyperplasia has been observed following treatment regression after PRK^26^,^27^. Also, stromal tissue deposition correlated with postoperative regression in PRK patients^26^. Healing of corneal epithelium and stromal remodeling could add uncertainty to refractive outcomes and effectiveness of astigmatism correction. Comparative analysis of epithelial and stromal remodeling between the 2 procedures is warranted to support our hypothesis. Adjunctive mitomycin C has been demonstrated to prevent post-PRK corneal haze, especially for high myopic correction^28^. Mitomycin C could delay healing and induce apoptosis of anterior stromal cells, modifying the healing response^29^,^30^. However, high variance in postoperative spherical equivalent was found after mitomycin C application during PRK compared to PRK alone for low to moderate ablation^31^. We were unsure if mitomycin C affected predictability of astigmatic correction in PRK in our cohort.

Our study results did not include the impact of SMILE and PRK on corneal biomechanics. The SMILE procedure has potential benefit over flap-based surgeries in terms of biomechanical stability. Mathematical models have predicted that the tensile strength after SMILE is better compared to PRK and LASIK^2^. Dou *et al*. showed that although both SMILE and Laser-assisted subepithelial keratomileusis alter corneal biomechanical strength, the changes induced by SMILE are more predictable than those induced by LASEK in terms of per unit tissue removed^9^. Further research is warranted to evaluate the effect of these changes on visual and refractive results after SMILE and PRK.

The current study was limited by a small sample size and non-randomized design. We did not have access to keratometry, epithelial thickness maps and contrast sensitivity values. We also acknowledge the relatively short follow-up duration, which precludes analysis of the long-term stability of our results. However, significant group difference in astigmatism correction was detected between SMILE and PRK in vector analysis during the early postoperative period. In conclusion, both SMILE and PRK were observed to be safe for correction of low to moderate astigmatism in eyes with high myopia. Predictability of refraction was superior in SMILE, which showed a better treatment alignment of astigmatism than PRK in vector analysis. There was a trend towards overcorrection of cylindrical correction following surface ablation.

## Methods

This study was a prospective audit of consecutive patients who received SMILE and PRK with mitomycin C at the Hong Kong Laser Eye Center and Chinese University of Hong Kong Eye Centre respectively. Patients were offered either PRK or SMILE in the respective surgery centres. All surgeries were performed between June 2014 and May 2015. The Joint Chinese University of Hong Kong-New Territories East Cluster Clinical Research Ethics Committee approved the study protocol. An informed consent was obtained from all study participants. The study adhered to the tenets of the Declaration of Helsinki.

Patients with no ocular abnormality except myopia and myopic astigmatism, a corrected distance visual acuity (CDVA) of 20/20 or better, stable refraction for more than 1 year, manifest myopic refraction spherical equivalent of ≥6.0 D and astigmatism between −0.25 D and −2.50 D were included. All eyes had emmetropia as the target refraction. Patients with corneal thickness of <500 μm, suspicion of keratoconus on corneal topography (displacement of the corneal apex, decrease in thinnest-point pachymetry, asymmetric topographic pattern), ocular inflammation, infection were excluded.

### Small incision lenticule extraction

SMILE was performed using the 500-kHz VisuMax femtosecond laser (Carl Zeiss Meditec, Jena, Germany) using a standard surgical technique (Sekundo BJO 2011). The following parameters were used: cap thickness, 120 μm; cap diameter, 7.5 mm; lenticule diameter, 6.5 mm with a transition zone of 0.1 mm; cut energy, 1.4 μJ; spot and tracking distance, 2.0–3.0 μm. The back of the intrastromal lenticule was created from periphery to center of cornea. The anterior lamellar cut was subsequently created from center to periphery of cornea, which extended towards the surface to create a 2–3 mm incision, from which the stromal lenticule was extracted. A thin blunt spatula was used to separate the lenticule, which was then grasped with a pair of forceps and removed. The corneal interface was flushed with balanced salt solution.

### Photorefractive keratectomy with mitomycin C

Alcohol-assisted corneal epithelial removal was performed over a 9.0-mm optical zone centered over the pupil. Ethanol 20% in distilled water was dropped into a 9.0-mm well and kept in contact with the epithelium for 40 seconds and then absorbed with a dry cellulose sponge. The eye was then washed with Balanced Salt Solution. A blunt spatula was then used to remove the loosened epithelium. Stromal ablation was performed with Allegretto Wave & Eye-Q 400Hz laser (WaveLight Laser Technologie AG, Germany) using a 6.5 mm optical zone. The system has an infrared high-speed camera operating to track the patient’s eye movements that either compensates for changes in eye position or interrupts the treatment if the eye moves outside a preset predetermined range. After the stromal ablation, a circular cellulose sponge soaked with mitomycin C 0.02% (0.2 mg/mL) was placed on the cornea for 60 seconds. The eye was then copiously irrigated with a balanced salt solution to remove the residual mitomycin C. A bandage contact lens was placed over the cornea at the end of the surgery.

Patients were examined preoperatively and 1, 3 and 6 months postoperatively. Severity of corneal haze after PRK was evaluated by slit-lamp biomicroscopy according to Hanna’s scale^32^. It was graded as follows: 0 = clear cornea, no haze; 0.5 = barely perceptible, seen only by tangential illumination; 1 = trace haze of minimal density seen with difficulty using direct illumination; 2 = moderate haze easily visible with direct slit illumination; 3 = marked haze that partially obscures anterior chamber observation or iris detail; 4 = severe haze that obscures anterior chamber or iris details^33^. Uncorrected visual acuity (UCVA), corrected distance visual acuity (CDVA) and manifest refraction were analyzed. Efficacy index was calculated as the ratio of postoperative UCVA to preoperative CDVA. Safety index was expressed as the ratio of postoperative CDVA to preoperative CDVA.

### Vector analysis

Refractive astigmatism at the spectacle plane was converted to corneal plane using vertex distance of 12 mm. It was then analyzed using vector analysis, with consideration of change in the astigmatic axis, measuring 3 vectors and relationship among them (Alpins JCRS 2001). The target induced astigmatism vector (TIA) was defined as the astigmatic change which the surgery was intended to induce; the surgically induced astigmatism vector (SIA) was defined as the astigmatic change which the surgery actually induced; and the difference vector (DV) was defined as the induced astigmatic change that would enable the initial surgery to achieve its intended target or the postoperative astigmatism. Magnitude of error (ME) is the arithmetic difference between the SIA and TIA. Angle of error (AE) is the angle between the axis of the SIA and TIA.

### Statistical Analysis

Statistical analysis was performed using R 2.15.3 (R Foundation, Vienna, Austria). Visual and refractive outcomes, efficacy and safety index were evaluated at different time points. Linear mixed effect models were used to adjust for correlation between fellow eyes. Comparisons of mean values were performed using repeated measures analysis of variance (ANOVA) at each time point. A p-value < 0.05 was considered as statistically significant. Bonferroni adjustments were considered for multiple testing.

## Additional Information

**How to cite this article**: Chan, T. C. Y. *et al*. Early outcomes after small incision lenticule extraction and photorefractive keratectomy for correction of high myopia. *Sci. Rep.* **6**, 32820; doi: 10.1038/srep32820 (2016).

## Figures and Tables

**Table 1:** Comparison of visual and refractive outcomes between small-incision lenticular extraction and photorefractive keratectomy.

		SMILE	PRK	P-value
CDVA (logMAR)	Pre-Op	0.001 ± 0.008	−0.018 ± 0.029	0.008
	Month 1	0.027 ± 0.041	0.039 ± 0.091	0.581
	Month 3	0.018 ± 0.029	0.011 ± 0.050	0.600
	Month 6	0.021 ± 0.055	0.013 ± 0.049	0.594
UDVA (logMAR)	Month 1	0.051 ± 0.069	0.133 ± 0.129	0.009
	Month 3	0.061 ± 0.081	0.070 ± 0.085	0.732
	Month 6	0.044 ± 0.058	0.060 ± 0.093	0.452
Efficacy Index	Month 1	0.903 ± 0.131	0.735 ± 0.200	0.002
	Month 3	0.886 ± 0.146	0.833 ± 0.171	0.313
	Month 6	0.915 ± 0.115	0.858 ± 0.193	0.215
Safety Index	Month 1	0.947 ± 0.087	0.892 ± 0.164	0.188
	Month 3	0.965 ± 0.066	0.942 ± 0.122	0.463
	Month 6	0.962 ± 0.099	0.940 ± 0.125	0.537
Spherical	Pre-Op	−7.37 ± 0.72	−7.65 ± 0.99	0.326
Equivalent (D)	Month 1	0.00 ± 0.28	−0.43 ± 0.35	0.000
	Month 3	−0.05 ± 0.29	−0.27 ± 0.27	0.011
	Month 6	−0.12 ± 0.26	−0.28 ± 0.28	0.044
Sphere (D)	Pre-Op	−6.77 ± 0.82	−7.10 ± 1.01	0.277
	Month 1	0.07 ± 0.32	−0.09 ± 0.19	0.029
	Month 3	0.04 ± 0.29	−0.03 ± 0.12	0.228
	Month 6	−0.01 ± 0.25	−0.04 ± 0.18	0.680
Cylinder (D)	Pre-Op	−1.20 ± 0.58	−1.10 ± 0.66	0.625
	Month 1	−0.16 ± 0.23	−0.70 ± 0.59	0.001
	Month 3	−0.19 ± 0.23	−0.47 ± 0.53	0.037
	Month 6	−0.21 ± 0.21	−0.48 ± 0.45	0.014

SMILE = small-incision lenticule extraction, PRK = photorefractive keratectomy, CDVA = corrected distance visual acuity, Pre-Op = preoperative, UDVA = uncorrected distance visual acuity.

**Table 2:** Vector analysis of astigmatic correction at 6 months after small-incision lenticule extraction and photorefractive keratectomy using Alpins method.

		SMILE	PRK	P-value
TIA	Arithmetic mean ± SD (D)	1.05 ± 0.46	0.92 ± 0.54	0.235
	Range^a^ (D)	1.02 to 1.07	0.89 to 0.96	
SIA	Arithmetic mean ± SD (D)	1.08 ± 0.43	1.26 ± 0.78	0.704
	Range^a^ (D)	1.05 to 1.10	1.22 to 1.31	
DV	Arithmetic mean ± SD (D)	0.20 ± 0.20	0.48 ± 0.44	0.005
	Range^a^ (D)	0.19 to 0.22	0.45 to 0.51	
ME	Arithmetic mean ± SD (D)	0.03 ± 0.22	0.34 ± 0.47	0.001
	Range^a^ (D)	0.02 to 0.04	0.31 to 0.37	
Absolute AE	Arithmetic mean ± SD (°)	5.0 ± 9.4	9.8 ± 13.6	0.040
	Range^a^ (°)	4.5 to 5.6	9.0 to 10.7	
CI	Geometric mean ± SD	1.02 ± 0.28	1.34 ± 0.86	/
	Range^a^	1.00 to 1.04	1.29 to 1.40	
FI	Geometric mean ± SD	0.78 ± 0.88	0.90 ± 1.22	/
	Range^a^	0.73 to 0.83	0.83 to 0.97	
IOS	Geometric mean ± SD	0.13 ± 0.31	0.48 ± 0.96	/
	Range^a^	0.12 to 0.15	0.42 to 0.53	

SMILE = small-incision lenticule extraction; PRK = photorefractive keratectomy; AE = angle of error; CI = correction index; DV = difference vector; FI = flattening index; IOS = index of success; SIA = surgically induced astigmatism; TIA = target-induced astigmatism.

^a^95% confidence interval.
